# Elucidating the role of autoreactive T cells and B cells in autoimmune hepatitis

**DOI:** 10.1172/JCI188538

**Published:** 2025-01-16

**Authors:** Yoshiaki Yasumizu, David A. Hafler

**Affiliations:** Departments of Neurology and Immunobiology, Yale School of Medicine, New Haven, Connecticut, USA

## Abstract

How are autoreactive T cells induced and regulated in patients with autoimmune disease? This question lies at the core of understanding autoimmune disease pathologies, yet it has remained elusive due to host variability and the complexity of the immune system. In this issue of the JCI, Kramer and colleagues used autoimmune hepatitis (AIH) as a model to explore the maintenance of autoreactive CD4^+^ T cells specific to O-phosphoseryl-tRNA:selenocysteine tRNA synthase (SepSecS). The findings provide insight into the interaction between T cells and B cells in AIH pathogenesis that may reflect a shared mechanism among other autoimmune diseases.

## Pathogenesis of autoimmune hepatitis

Autoimmune hepatitis (AIH) is an organ-specific autoimmune disease that has been characterized by autoantibodies, in particular those targeting O-phosphoseryl-tRNA:selenocysteine-tRNA synthase (SepSecS), also known as soluble liver antigen (SLA), in a subset of patients. The disease is characterized by elevated IgG levels, plasma cell infiltration in the liver, interface hepatitis, and responsiveness to steroids. Further evidence of an autoimmune pathophysiology is provided by a strong association with HLA class II molecules, suggesting a genetic predisposition involving CD4^+^ T cells. Finally, both the presence of autoantibodies, as discussed above, and plasma cell infiltration in liver lesions have suggested the involvement of B cells in AIH, though their role in the disease has not been known (1). Moreover, there is growing evidence supporting the hypothesis that liver damage is primarily driven by autoreactive T cells (2). Thus, it raises the question as to the role of B cells and T cells in AIH.

## Autoantibodies in autoimmune hepatitis

**Figure 1:**
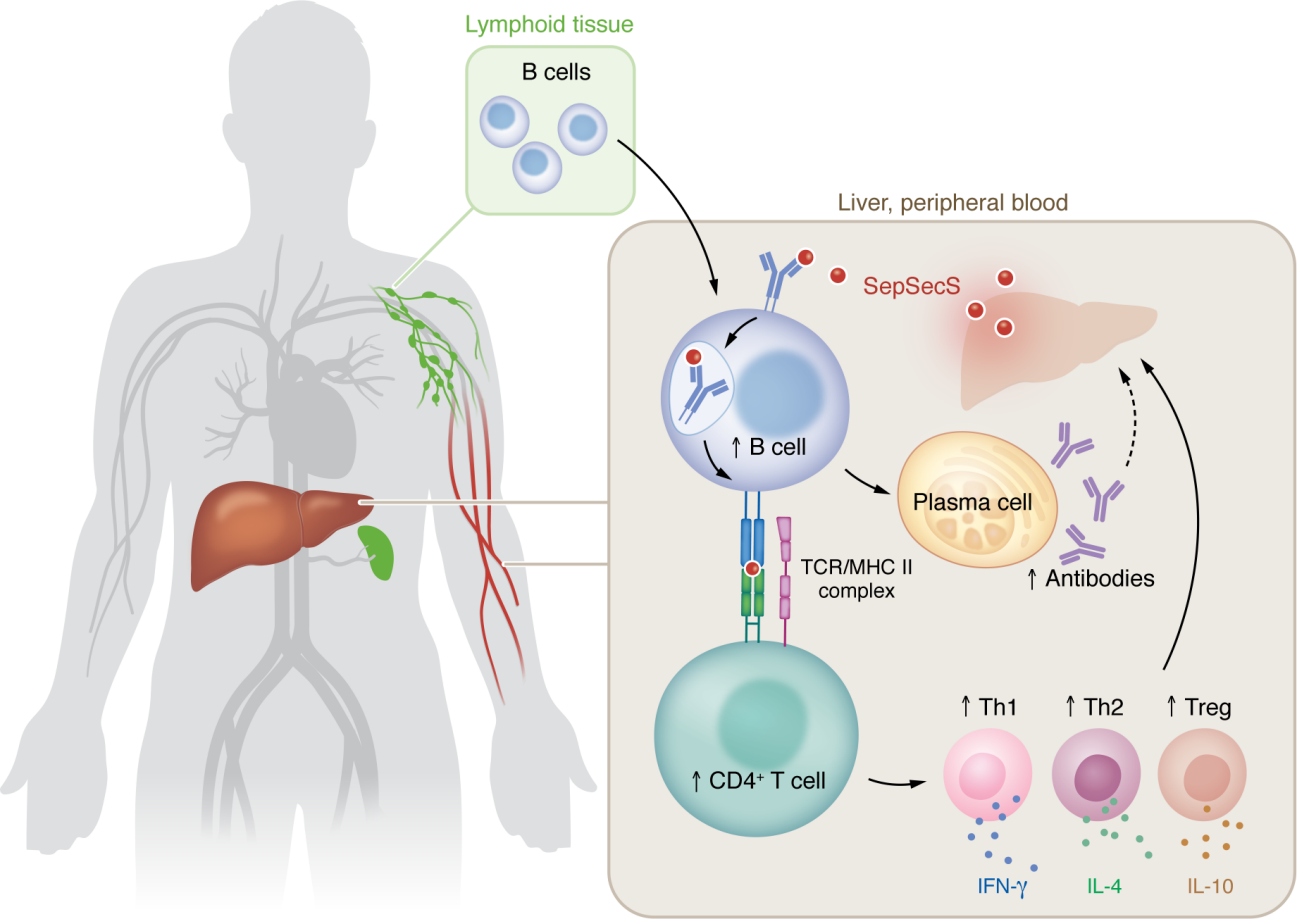
B cells orchestrate autoreactive CD4^+^ T cells in AIH. SepSecS-specific B cells present antigens to autoreactive CD4^+^ T cells via MHC class II molecules. Liver-infiltrating SepSecS-specific CD4^+^ T cells secrete IL-10, IL-4, and IFN-γ. The pathways may have a central role in the pathogenesis of AIH by driving the generation of pathogenic T cells that contribute to liver tissue damage.

In this issue of the JCI, Kramer et al. (3) addressed the role of B cells using an elegant, high-throughput profiling technique for antigen-specific B and T cells. They first demonstrate that SepSecS-specific antibodies and memory B cells were elevated in the peripheral blood of patients with AIH. Additionally, the affinity of B cell receptors (BCRs) for the SepSecS antigen increased through somatic hypermutation, suggesting that SepSecS-reactive B cells underwent maturation in germinal centers. It was also found that SepSecS-reactive CD4^+^ T cells were elevated in the blood and infiltrated the liver in the one patient with AIH where samples were available. This finding implies that these CD4^+^ T cells may act as T follicular helper (Tfh) cells aiding B cells (4) or as Th1 cells, which have been reported to cause liver damage (5). Importantly, Kramer et al. revealed that SepSecS-specific memory B cells could provide help to SepSecS-specific CD4^+^ T cells, indicating that B cells actively function as antigen-presenting cells (APCs) to maintain autoreactive CD4^+^ T cells in AIH (Figure 1).

## Role of autoreactive T cells in autoimmune hepatitis

SepSecS-specific CD4^+^ T cell clones were predominantly found in patients with AIH who were anti-SLA positive and anti-SLA negative. To a lesser extent, SepSecS-specific CD4^+^ T cell clones were also found in pathological and healthy controls, though SLA-positive patients possessed clones that recognized a great diversity of epitopes, perhaps suggesting epitope spreading with the chronic liver inflammation. Moreover, these clones secreted IFN-γ, IL-4, and IL-10. While this profile may seem surprising, a fundamental observation in organ-specific autoimmune diseases in humans has been the presence of organ-specific autoreactive T cells in patients as well as in healthy participants. As observed in Kramer et al. and other studies, autoreactive T cells are found at a modestly higher frequency in patients with autoimmune disease. Moreover, T cell library approaches in other autoimmune diseases indicate that autoreactive T cells are more inflammatory (6–8), while IL-10–secreting autoreactive T cells may be immunoregulatory. Recent studies in CNS tissue damage have suggest that autoreactive T cells may have a role in tissue repair (9), providing a theoretical framework for observations of healthy participants in which autoreactive T cells are poised physiologically to repair damaged organs.

## B cells as antigen presenting cells in autoimmune hepatitis

Among professional APCs, it was discovered back in 1985 by Lanzavecchia and colleagues that B cells are unique, as they utilize B cell receptors (BCRs) to uptake antigens, allowing highly selective antigen presentation and epitope spreading (10, 11). B cells recognize and efficiently capture and present antigens, which contribute to T-cell differentiation (12–14). In addition, memory B cells possess essential components for modulating T cell function, including expression of HLA class II, costimulatory molecules such as CD80/86, and cytokines such as GM-CSF and IL-6 (15). The selectivity of antigens can be further fine tuned through somatic hypermutation, depending on the antigen loads. Clinically, anti-CD20 antibody–based B cell depletion therapy has been reported as effective in AIH (16). This therapy eliminates naive and memory B cells, while antibody-producing plasma cells often remain due to low CD20 expression. Kramer et al.’s findings suggest that memory B cells play a crucial role as APCs in AIH and may acquire even more robust functionality through somatic hypermutation. Furthermore, the therapeutic efficacy may be attributed to the elimination of these memory B cells acting as APCs. Memory B cells possess the ability to differentiate into long-lived plasma cells (17), potentially linking to the increase of autoantibodies in AIH.

## Shared features with other organ-specific autoimmune diseases

The findings from Kramer et al. (3) suggest that T cell modulation may be a shared feature across other autoimmune diseases. MS is another autoimmune disease where B cell depletion is effective, and changes in CD4^+^ T cells have been observed postdepletion (18). Moreover, it is notable that B cell–mediated presentation of self antigens has been reported to generate pathogenic T cells, such as Th1 and Th17, in the context of MS (19, 20). Similarly, B cells have been implied to act as APCs to activate T cells and contribute to pathogenesis in other diseases such as rheumatoid arthritis (RA) and Sjögren’s syndrome (21). In these diseases, the encounter between autoreactive T and B cells may generate a synergistic spiral of mutual differentiation, proliferation, and increase of self-antigen release by tissue damage. Kramer et al. (3) sheds light on this spiral, offering insights and methodologies applicable to other autoimmune diseases.

## Future directions

Further investigation of AIH is necessary to elucidate why homeostasis fails. Negative selection in the thymus and regulatory T cells (Tregs) in peripheral tissues maintain immune homeostasis by preventing autoreactivity. AIH is thought to be influenced by genetic factors — including the HLA region — as well as environmental factors — such as viral infections and gut microbiome changes (22). Genome-wide association studies in White participants have also implicated CD28/CTLA4/ICOS regions (23), suggesting that abnormalities in costimulatory molecules may lower the threshold for autoreactivity.

Kramer et al. (3) deeply investigated T cell–B cell interactions with a focus on the specificity of a single antigen. Further investigations to elucidate the phenotype of SepSecS-specific cells using high-throughput systems combining antigen screening with single-cell RNA-seq may enable the deeper profiling of antigen-TCR/BCR-gene expression. Computational approaches, such as network analysis and machine learning models (24, 25), have been established to comprehensively explore antigen recognition and variable regions. These technologies may offer clues on the presence and maintenance of autoreactive lymphocytes in patients. In some conditions, autoreactive T cells are reported to evade exhaustion and maintain a stem-like phenotype (26), functioning as a reservoir for effector T cells. The molecular biological methods established by Kramer et al. (3) could lay the groundwork for phenotyping autoreactive lymphocytes. In summary, Kramer et al. (3) highlights the dynamics of autoreactive immune cells in AIH, revealing T and B cell interactions in AIH pathogenesis. Elucidating the mechanisms underlying immune homeostasis breakdown in AIH will hopefully lead to more effective and sustainable treatments.

## Acknowledgment

YY is supported by JSPS Overseas Research Fellowships. DAH is supported by NIH grants (P01 AI073748, U19 AI089992 U24 AI11867, R01 AI22220, UM 1HG009390, P01 AI039671, P50 CA121974, R01 CA227473) and Race to Erase MS and National MS Society to DAH.

## Version 1 01/16/2025

Electronic publication
